# Is explantation of silicone breast implants useful in patients with complaints?

**DOI:** 10.1007/s12026-016-8813-y

**Published:** 2016-07-13

**Authors:** M. de Boer, M. Colaris, R. R. W. J. van der Hulst, J. W. Cohen Tervaert

**Affiliations:** 1 Faculty of Health, Medicine and Life Sciences, Maastricht University, Maastricht, The Netherlands; 2 Reconstructive, Plastic and Hand Surgery, Maastricht University Medical Center, Maastricht, The Netherlands; 3 Clinical and Experimental Immunology, Reinaert Clinic, Maastricht, The Netherlands

**Keywords:** Silicone breast implants, Silicone (adverse effect), Explantation, Removal

## Abstract

In this review, we present a critical review of the existing literature reflecting the results of explantation of silicone breast implants in patients with silicone-related complaints and/or autoimmune diseases. A literature search was performed to discuss the following issues: which clinical manifestations and autoimmune diseases improve after explantation, and what is the course of these complaints after explantation. Next, we reviewed studies in which the effect of explantation on laboratory findings observed in patients with silicone breast implants was studied, and lastly, we reviewed studies that described the effect of reconstruction of the breast with a new implant or autologous tissue after explantation. We calculated from the literature that explantation of the silicone breast improved silicone-related complaints in 75 % of the patients (469 of 622). In patients with autoimmune diseases, however, improvement was only infrequently observed without additional therapy with immunosuppressive therapy, i.e., in 16 % of the patients (3 of 18). The effect of explantation did not influence autoantibody testing such as ANA. We discuss several possibilities which could clarify why patients improve after explantation. Firstly, the inflammatory response could be reduced after explantation. Secondly, explantation of the implants may remove a nociceptive stimulus, which may be the causative factor for many complaints. Options for reconstruction of the explanted breast are autologous tissue and/or water-/hydrocellulose-filled breast implant. Unfortunately, in very few studies attention was paid to reconstructive possibilities. Therefore, no adequate conclusion regarding this issue could be drawn. In conclusion, explantation is useful for improvement of silicone-related complaints in 75 % of the patients, whereas in patients who developed autoimmune diseases improvement is only observed when explantation is combined with immunosuppressive therapy. In a patient with silicone-related complaints in which explantation is considered, the patient should be counseled for the different options of reconstruction after explantation.

## Introduction

Since the introduction of silicone breast implants in the early 1960s, it has been postulated that patients may develop complaints related to silicone breast implants. Hence at present, there is still controversy whether breast implants are safe [1, 2].

Patients with implants may develop nonspecific complaints such as arthralgia, myalgia and fatigue. In the past years, these complaints in patients with silicone breast implants have been named differently: human adjuvant disease or adjuvant breast disease, silicone-related symptom complex, siliconosis and more recently ASIA syndrome due to silicone implant incompatibility syndrome (SIIS) [3–11]. In these patients, it is postulated that silicone act as an adjuvant to the immune system, resulting in inflammation, autoimmune diseases, immunodeficiencies and/or allergies [10]. In search of an effective therapy for these patients, it is the current practice to advise patients to undergo explantation of their implants. In this paper, we review the existing literature addressing the effectiveness of implant removal as treatment for patients with complaints that are possibly related to their silicone breast implants.

## Methods

### Study selection

We performed a comprehensive literature search in PubMed, MEDLINE, EMBASE and the Cochrane Central Register of Controlled Trials, and the Cochrane Database of Systematic Reviews through the first quarter of 2016. Additional citations were solicited from references in selected articles. The searches combined the following terms: ‘Breast implants [Mesh],’ ‘Silicone, adverse effect [Mesh],’ for the period between January 1960 and the present time. An additional search to cross-reference the outcome of the previous search was performed with the terms ‘removal,’ ‘explantation’ and ‘Device Removal Mesh.’

We included studies discussing patients with breast implants (silicone- or saline-filled) who reported or presented with silicone-related complaints (Table 1) after insertion of the breast implants and who underwent explantation of the breast implant [4, 6–9]. Studies of all type were included, meaning case reports, case series, case–control studies and descriptive cohort studies. We excluded studies that described explantation of breast implants that was performed because the implants were ruptured and/or were leaking and no description of silicone-related complaints was mentioned. We excluded also studies focussing on malignancies of the breast after silicone breast implantation. Also, studies focussing on silicone oil/gel injections were excluded.

**Table 1:** Silicone-related complaints and other silicone-related manifestations

Silicone-related complaints	Fatigue
	Myalgia
	Arthralgia
	Pyrexia
	Sicca (dry eyes/dry mouth)
	Memory, concentration and sleep disturbances
	Neurological manifestations (TIA/CVA, demyelinisation)
Other	Raynaud’s phenomenon
	Irritable bowel syndrome
	Allergies
	Immunodeficiencies
	Autoimmune diseases

We report on: (1) whether improvement of several silicone-related complaints (including autoimmune diseases) after explantation (Table 1) occurred; (2) what the course of improvement of complaints is after explantation; (3) what the effect of explantation is on laboratory findings; (4) whether patients underwent reconstruction of the breast after explantation or not.

### Study extraction and assessment

Data from each included study were extracted. Extracted data included study type, participants, implant characteristics (if available), silicone-related complaints (Table 1), laboratory findings (if available), explantation, status of silicone-related complaints/autoimmune disease after explantation, status of laboratory findings after explantation (if available), course of disease/symptoms after explantation, reconstruction of explanted breast (if available).

**Fig. 1:**
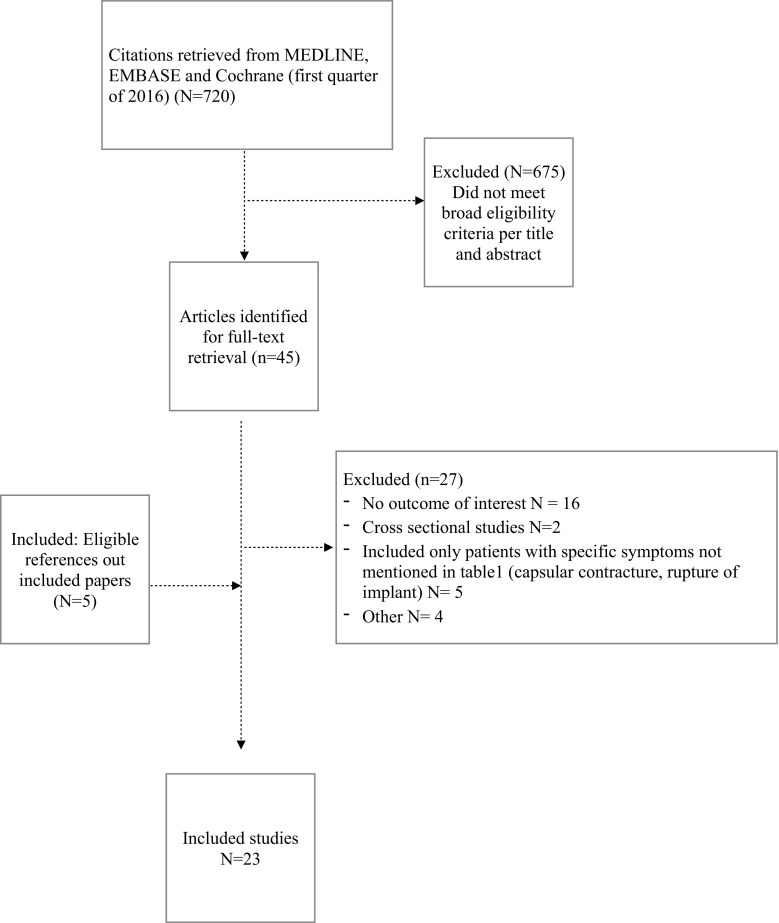
Summary of evidence search and selection

The literature search yielded 720 citations. Firstly, titles and abstracts were read. On the basis of titles and abstracts, 45 publications were provisionally accepted for review. After screening of the full text, 17 studies met eligibility criteria. Additionally, 6 studies were selected based on references in these 17 studies that also met eligibility criteria and were therefore retrieved and used as well (Fig. 1). Of the 23 included studies, 10 were case reports/case series (Table 2) and 13 studies were cohort studies (Table 3).

**Table 2:** Summary of case reports

References	Reason insertion SBI	Silicone-related complaint	Presence of autoimmune disease	Laboratory findings	Intervention	Outcome
Teuber et al. [12]	Cosmetic	Raynaud, myalgia, pyrexia, malaise, lymphadenopathy	Sarcoidosis		Explantation, prednisone	Clinical improvement, resolvement cutaneous sarcoidosis and lymph nodes
Kivity et al. [13]	Cosmetic	Morphea, myalgia, scleroderma-like lesions			Explantation, oral 1 mg/kg prednisone	Minor improvement in myalgia and morphea, due to prednisone
Chan et al. [14]	Cosmetic	Arthralgia, fatigue	–	Elevated ESR, positive ANA, IgG cardiolipin antibody	Explantation, MTX and prednisone	Complete solution of symptoms and laboratory findings
Nesher et al. [5]	Reconstruction	Arthralgia, fatigue, myalgia, sicca, hand paresthesia	–		Explantation	No improvement in symptoms
Kappel et al. [15]	Reconstruction	Fatigue, arthralgia, myalgia, sleep disturbances	–		Explantation	Full improvement in all symptoms in three sisters
Jara et al. [16–19]	Cosmetic/reconstruction	Arthritis, fever, myalgia, conjunctival hyperemia, odynophagia	Still’s disease		Explantation, steroids (IVIG, AZA or MTX)	Remission of Still’s disease, 3 of 4 patients steroid dependent
Levy et al. [20]	Reconstruction	1. Raynaud, fibrotic skin, swollen digiti 2. Raynaud, arthralgia, sclerodactyly	1. Scleroderma 2. Scleroderma	1. Positive ANA + anti-ScL-70 2. Increased CRP, positive ANA	Explantation	1. Disease progression, no change in laboratory screening 2. No improvement, increased CRP, strong + ANA, anti-centromere antibodies, anti-DNA
Granel et al. [21]	Reconstruction	Morphea		–	Explantation, replacement with saline-filled implant with polyurethane capsule	Disease progression with persisting morphea
Meier et al. [22]	Reconstruction	Neurological manifestations, arthritis	Polyarthritis	–	Explantation	1 Complete remission, 2 mild residual symptoms
Homsi et al. [23]	Reconstruction	Digital ischemia, right leg weakness, inflammation left breast	Polyarteritis nodosa	Increased CRP	Explantation, steroids and mycophenolate mofetil	Persistent remission
Shoaib and Patten [24]	Cosmetic	Arthralgia, fatigue, neurological complaints, myalgia, memory and concentration problems, sicca complaints, IBS and Raynaud’s phenomena	(atypical) Multiple sclerosis	–	Pt 1: Explantation + immunosuppressive therapy Pt 2: Explantation	Pt 1: Improvement, after immunosuppressive therapy Pt 2: Diminished Raynaud

*ESR* erythrocyte sedimentation rate, *ANA* antinuclear antibody, *IVIG* intravenous immunoglobulins, *AZA* azathioprine, *MTX* methotrexate, *CRP* C-reactive protein

**Table 3:** Summary of cohort studies

References	Number of patients	Silicone-related complaint (s)	Presence of autoimmune disease	Intervention	Outcome of explantation
Vasey et al. [25]	*N* = 33	Chronic fatigue, myalgia, arthralgia, lymphadenopathy	–	Explantation	24 patients total improvement, 8 no improvement, 1 disease progression
Aziz et al. [26]	*N* = 43	Arthralgia, myalgia, fatigue	–	Explantation	Improvement in 97 %
Thomas et al. [27]	*N* = 25	Arthralgia, fibromyalgia, sicca, hypesthesia	–	Explantation	Improvement in 25 patients
Kappel and Pruijn [28]	*N* = 22+ *N* = 13+	Myalgia, fatigue, arthralgia, memory/sleep disturbances	–	Explantation + replacement with hydrocellulose filled implant (+ capsulectomy)	Significant decline in all mentioned symptoms (except arthralgia) in all patients (*n* = 35)
Walden et al. [29]	*N* = 22	Arthralgia, skin lesions	–	Explantation	Improvement of complaints in all patients
Rohrich et al. [30]	*N* = 38	Arthralgia, pain and fatigue	–	Explantation	Improvement in skeletal symptoms, bodily pain, vitality, mental health and body image
Svahn et al. [31]	*N* = 63	General strength, vitality, arthralgia, pain and memory	–	Explantation	Improvement in quality of life in 78 % of patients
Melmed [32]	*N* = 240	Fatigue, memory loss, arthralgia, dysphagia, sicca depression, altered sleep, hair loss, skin rash, headache, neurological manifestations	–	Explantation	Improvement in 74 % of patients, especially sicca, flu-like symptoms. Neurological manifestations did not improve
Godfrey and Godfrey [33]	*N* = 37	Fatigue, myalgia, arthralgia, hair loss, paresthesia, Raynaud’s, frequent infections, dry eyes/mouth, dizziness, headache	–	Explantation + TRAM flap/latissimus dorsi flap	Improvement in 89.2 % patients. Steady return of complaints, to only 32.4 % improved patients after 6 months
Peters et al. [34]	*N* = 75	Arthralgia, myalgia and breast pain	SLE *N* = 2, MS *N* = 1, RA *N* = 2, Raynaud’s disease *N* = 1	Explantation	Improvement in 74 % of patients (*n* = 56). No improvement in patients with a autoimmune disease
Maijers et al. [35]	*N* = 52	Fatigue, neurasthenia, arthralgia, myalgia, morning stiffness, night sweats, dyspnoea, cognitive impairment, dermatological symptoms, disorders of the digestive tract and alopecia	CTD *N* = 5, IBD *N* = 2, Other *N* = 7	Explantation	Improvement in 70 % of the patients
Campbell et al. [36]	*N* = 40	Suppressed natural killer cell activity	–	Explantation	Resolvement of natural killer cell activity in 50 % patients

*TRAM-flap* transverse rectus abdominus myocutaneous-flap, *SLE* systemic lupus erythematosus, *MS* multiple sclerosis, *RA* rheumatoid arthritis, *CTD* connective tissue disease, *IBD* inflammatory bowel disease

## Results

### Improvement of complaints after explantation: case reports

Teuber et al. described a 45-year-old woman who presented with Raynaud’s phenomenon, myalgia, pyrexia, malaise, cutaneous lesions, uveitis, enlarged lymph nodes and shortness of breath, which developed 7 years after cosmetic mamma augmentation. A X-ray of the chest showed bilateral hilar lymphadenopathy and a transbronchial biopsy revealed noncaseating granulomas consistent with sarcoidosis. The course of the sarcoidosis was progressive and only minimal responsive to prednisone (minimal improvement in pulmonary, ocular and joint symptoms). However, cutaneous sarcoidosis and enlarged lymph nodes resolved after explantation, whereas symptoms and clinical condition improved dramatically as well [12].

Kivity et al. presented a patient who developed myalgias and morphea after breast augmentation with silicone breast implants. Due to tightening of the skin around the implants and significant discomfort, the implants were surgically removed. The clinical symptoms (myalgia, morphea) did not improve after implant removal, whereas treatment with 1 mg/kg prednisone resulted in some improvement [13].

Chan et al. described a patient with arthralgias and fatigue, which developed after mamma augmentation with silicone breast implants 7 years earlier. Laboratory screening showed increased inflammatory markers, such as elevated sedimentation rate, positive ANA and IgG anti-cardiolipin antibodies. A diagnosis of an unspecified inflammatory disease was made, and treatment with methotrexate and steroids was started. Ultrasound of the breast showed a ruptured left breast implant. The patient chooses to replace the breast implants by new silicone gel-filled implants. Soon after surgery, she developed a rash. Subsequently, her breast implants were removed. 10 weeks later, the methotrexate and prednisolone could be stopped and the patient showed complete resolution of her symptoms and the inflammatory response [14].

Nesher et al. presented a patient with a silicone breast implant placed for reconstruction after a mastectomy. After implant rupture a revision with a new silicone implant was performed and subsequently, the patient developed fatigue, arthralgia, myalgia, dry eyes, cognitive impairment, intermittent abdominal pain, attacks of fainting, weight loss, headaches and hand paresthesias [5]. An MRI showed enlarged supra-clavicular lymph nodes possibly due to silicone granuloma and enhancement of the chest wall after gadolinium injection. After explantation, the fibromyalgia-like symptoms did not improve, whereas generalized weakness, fatigue and insomnia also persisted.

Kappel et al. described three sisters with a BRCA-1 gen mutation who underwent preventive mastectomy and reconstruction with silicone breast implants. All three women developed fatigue, arthralgias, myalgias and sleep disturbances within a period of four years after implantation [15]. All complaints improved as evaluated 2.5 years after explantation of the implants.

Jara et al. presented a case report and discussed three other patients who developed Still’s disease after silicone breast implantation [16–19]. All four patients underwent implant removal and experienced improvement. However, all patients received additional therapy such as steroids, intravenous immunoglobulins, azathioprine or methotrexate. Three of the four patients remained steroid dependent during long-term follow-up.

Levy et al. presented two cases with silicone breast implants and systemic sclerosis who underwent explantation [20]. Case 1 was a patient who underwent bilateral mamma reconstruction with silicone breast implants. 14 years later, she developed Raynaud’s phenomenon, heartburn and swelling of fingers and toes, telangiectasia and fibrotic skin changes. Laboratory screening showed ANA and anti-ScL-70 autoantibodies. She underwent removal of the implants. Rupture occurred during removal and silicones entered into the blood stream. Several months later, she developed extreme dyspnea, severe restrictive lung disease with low CO diffusing capacity and interstitial lung disease. She died two years after implant removal from progressive systemic sclerosis. Case 2 comprises a 52-year-old patient who underwent mastectomy and 3 years later insertion of a silicone breast implant. 7 years later, she developed arthralgias, Raynaud’s phenomenon, sclerodactyly and telangiectasia. Laboratory screening showed increased CRP and positive ANA. Due to gradual hardening of the implant associated with pain, the patient opted for replacement. Several months later, the replaced implant ruptured and was removed without further replacement. Clinical symptoms did not improve after explantation.

Granel et al. presented a 53-year-old woman who underwent mamma reconstruction with silicone breast implants [21]. Localized morphea occured after 1 year. The implant was replaced by a saline-filled implant with a polyurethane-covered silicone capsule. Disease progression occurred with persisting morphea without signs of systemic sclerosis.

Meier et al. described two HLA identical sisters who both received silicone breast implants and subsequently developed polyarthritis and neurological symptoms [22]. After removal of the implants, the rheumatic as well as neurological symptoms improved dramatically in both patients. One patient achieved complete remission. The other patient had mild residual symptoms.

Homsi et al. presented a 49-year-old woman who presented with necrotizing vasculitis following silicone breast implants because of congenital breast asymmetry. The patients suffered from digital ischemia, right leg paresis and inflammation of the inferior part of the left breast. Due to cutaneous necrosis of the breast, the patient underwent capsulotomy and removal of the implant. Histopathological examination showed necrotizing arteritis. Treatment with high dose prednisone and mycophenolate mofetil was initiated, and after one year of follow-up, a persistent remission was observed [23].

Shoaib et al. described two patients with human adjuvant disease due to SBI who underwent implant removal [24]. The first patient presented with arthralgia, fatigue and neurological manifestations six years after (cosmetic) augmentation. She was diagnosed with atypical multiple sclerosis. Explantation did not result in improvement, whereas intravenous cyclophosphamide and immunoglobulins did. The second patient presented two years after augmentation with the same clinical manifestations as patient 1 and additional symptoms such as myalgia, memory and concentration problems, sicca complaints, irritable bowel syndrome and Raynaud’s phenomena. An MRI showed demyelinisation and she was diagnosed with atypical multiple sclerosis. She underwent explantation 3 years after the first symptoms and improvement was minimal, i.e., only observed in diminished Raynaud’s phenomenon.

Case reports are summarized in Table 2.

### Improvement of complaints after explantation: Case series

Vasey et al. presented 50 patients with silicone breast implants with findings such as fatigue, myalgias, arthralgias and lymphadenopathy [25]. Thirty-three women underwent implant removal and 17 did not undergo implant removal. During an observation period of 14 months, the complaints did not change in the 17 patients without explantation, whereas in the patients with explantation, 24 women improved (no symptoms anymore), 8 did not change, and in only one patient, symptoms worsened after an average follow-up of 22 months.

Aziz et al. prospectively followed 95 women who had silicone gel-filled breast implants and rheumatologic symptoms (arthralgia, myalgia) and fatigue and found that the symptoms improved in 42 (97 %) of the 43 women who had their breast implants removed [26]. In contrast, rheumatologic symptoms worsened in 50 (96 %) of 52 women who did not have their implants removed.

Thomas et al. presented 25 patients who underwent implant removal because of arthralgias, sicca complaints and hypesthesia [27]. Improvement in patient-reported symptoms and signs occurred over the course of months postoperatively in all patients.

Kappel et al. presented a study in which they compared a group of patients with silicone breast implants with complaints such as fatigue, myalgias, arthralgias, memory and sleep disturbances who underwent removal, capsulectomy and subsequently insertion of a hydrocellulose-filled implant (*n* = 22) to a group of patients with silicone breast implants with similar symptoms as the patients in the first group who underwent removal and insertion of a the hydrocellulose-filled implant, but no capsulotomy (*n* = 13) [28]. A questionnaire examining the presence of symptoms pre-operatively and post-operatively was filled in by patients of both groups. In both symptomatic groups, a significant decline of the presence of symptoms was observed. Only arthralgias, however, did not improve in the patients who underwent explantation without capsulectomy. Importantly, improvement appeared to be more pronounced when an additional capsulectomy was performed.

Walden et al. prospectively studied the outcome of explantation in a group of 22 patients with silicone implants with complains such as arthralgias using a questionnaire for health status and compared the results to a group of patients who underwent a cholecystectomy (*n* = 20) [29]. In the explantation group, self-reported health rating scores improved from 2.64 to 4.89, but did not change in the cholecystectomy group (7.57 to 8.07). Unfortunately, the exact number of patients who experienced improvement is not mentioned in this study.

Rohrich et al. prospectively assessed the efficacy of explantation of silicone breast implants in 38 women with complaints such as arthralgia and fatigue. Self-evaluation of the health status was done preoperatively and 6 weeks and 6 months postoperatively. In addition, the general practitioner evaluated the health status of the patients [30]. After explantation, patients showed an improvement in measurements of arthralgia and pain, as well as an increase in vitality, mental health and body area satisfaction when compared with preoperative measurements. Unfortunately, authors do not state what number of patients experienced improvement.

Svahn et al. retrospectively studied health improvement following removal of silicone gel-filled breast implants in 63 female patients [31]. Quality of life was assessed by a questionnaire in which physical and cognitive function was studied. Improvement in symptoms occurred in 49 of the 63 (78 %) patients regarding quality of life.

In the largest study to date, Mehmed et al. described explantation in 240 women who presented with symptoms such as chronic fatigue, memory loss, arthralgia, dysphagia, depression, altered sleep patterns, hair loss, skin rashes, headaches, flu-like symptoms and atypical multiple sclerosis [32]. After explantation, 74 % of the patients reported that they felt much better. Especially, dry eyes and flu-like symptoms improved quickly. MS-like symptoms, however, did not improve.

Godfrey et al. presented 37 patients with silicone breast implants and complaints such as fatigue, myalgia, arthralgia, hair loss, paresthesia, Raynaud’s phenomenon, dry eyes/mouth, dizziness and headache who underwent replacement of breast implants [33]. Postoperatively, a major improvement in symptoms was observed in 89 % of patients. However, at 6 months postoperatively, symptoms returned in most patients leaving only 32 % of the patients asymptomatic during longer follow-up.

Peters et al. evaluated the outcome of removal of breast implants in 75 symptomatic patients after extensive evaluation preoperatively [34]. Patients had complaints such as arthralgia, myalgia, fatigue, gastrointestinal symptoms, rashes, memory loss, sleep disturbances and breast pain. 2.7 years after explantation, 56 patients stated that their quality of life had improved. Six of 75 patients had a proven autoimmune disease (see Table 3). After follow-up, none of these patients had shown any improvement in clinical status or autoantibody levels.

Recently, Maijers et al. described a cohort of 80 patients presenting with complaints such as fatigue, arthralgias, myalgias, morning stiffness, night sweats, cognitive impairment, dermatological symptoms and/or alopecia. Fifty-two women underwent explantation. Thirty-six women reported a significant decrease in the symptoms, of which nine patients stated that they were completely without complaints [35]. Eleven patients had an autoimmune disease such as Sjögren’s syndrome or systemic sclerosis. Unfortunately, it is not described whether these patients did improve after explantation as well.

Case series are summarized in Table 3.

### Effect of explantation on laboratory findings

Kivity et al. presented a patient with morphea undergoing explantation of the breast implant. ANA was found to be positive before removal [13]. 4 weeks after removal, ANA were still present.

Also in the case that was reported by Jara et al., positive ANA were found before and after implant removal [16].

Levy et al. [20] reported a case with positive ANA. After explantation, ANA remained strongly positive, and anti-centromere antibodies and anti-dsDNA became detectable.

In the study by Kappel et al. [28], three sisters underwent explantation because of complaints. After explantation, IgG levels increased, whereas ANA remained positive in these patients.

Peters et al. presented 5 patients with autoimmune diseases and autoantibodies. After explantation (2.7 years) autoantibodies persisted.

Campbell et al. presented a study in which the NK cell function of 40 symptomatic patients with silicone breast implants was evaluated before and after explantation [36]. After explantation, NK activity increased in 50 % of the patients, whereas NK activity decreased in 26 % of the patients and was unchanged in 24 %. Unfortunately, no control group was studied to compare NK activity during follow-up in healthy controls.

### Effect of reconstruction after explantation

Few data are available with respect to the effect of reconstruction after explantation.

Granel et al. present a 53-year-old woman with morphea who underwent replacement of a silicone implant by a saline-filled implant with a polyurethane capsule. Disease progression occurred [21].

Kappel et al. describe explantation of silicone breast implants in three symptomatic sisters and subsequently replacement of hydrocellulose-filled implants [15]. Full recovery did occur.

In another study by Kappel et al. [28], patients underwent explantation of the silicone implant (with or without capsulectomy) and immediate reconstruction with a hydrocellulose-filled implant. Significant improvement in symptoms did occur in these patients as well (*N* = 35).

Godfrey et al. present 37 patients who underwent replacement of breast implants due to systemic complaints and subsequently reconstruction with autologous tissue (TRAM flap or latissimus dorsi flap) [33]. At 6 months postoperatively, only 32.4 % of the patients remained free of symptoms.

### Summary

#### Silicone-related complaints

In this review, we have described 11 case reports with a total of 19 patients who underwent explantation [12–24]. In these case reports, 12 of 19 patients (63 %) experienced improvement of their silicone-related complaints after explantation. Two of 17 patients experienced disease progression (12 %).

In the case series, we have 12 case series with a total of 703 patients who underwent explantation [25–36].

For 603 patients, it was well described whether clinical status improved after explantation [25–28, 31–35]. Of 603 patients, 457 (76 %) experienced improvement of silicone-related complaints after explantation. In one of 703 patients, evident disease progression after explanation was reported.

In total (case reports + case series), this implies that 469 of 622 patients (75 %) experienced improvement of silicone-related complaints after explantation.

#### Autoimmune diseases

If we only assess the improvement in autoimmune diseases after explantation, we see that in the case reports 10 of 12 patients with an autoimmune disease experienced some improvement after explantation [12, 16–19, 22–24]. However, 7 of 10 patients who improved after explantation also received immunosuppressive or immunomodulatory therapy before, during or after implant removal. In the case series, only one author, Peters [33], closely described the presence of patients with an autoimmune disease in his cohort and whether these patients improved after explantation. None of the 6 patients with well-defined autoimmune disease improved after explantation. In total, this implies that only 3 of 18 patients (16 %) with a well-defined autoimmune disease did improve after explantation without additional therapy. Furthermore, 7 of 18 patients (39 %) with autoimmune diseases improved after explantation in combination with adjuvant immunosuppressive therapy.

## Discussion

Whether silicone can elicit an inflammatory or autoimmune response has been subject of debate since the introduction of silicone breast implants. Up until the present, there is still no conclusive evidence that proves whether silicone implants are safe or unsafe. Furthermore, the exact prevalence of complaints in patients with silicone breast implants is unknown [1]. Several studies implicate that there is no increased risk to develop autoimmune diseases after silicone breast implant insertion [2]. Therefore, the FDA lifted the ban on these implants in 2004. Recently, it has been suggested that the methodological designs of the studies that influenced this decision were not correct and that more research, especially in larger prospective cohorts, is needed [3]. In light of the recent developments, e.g., the uproar concerning the PIP and Silimed prosthesis, the description of ASIA and breast implant-associated anaplastic large cell lymphoma (BIA-ALCL) [4, 37], we agree that more attention should be paid to silicone-related problems. Fortunately, this is already occurring as can be seen by the representation of the articles that were included in this review. After a first wave of articles in the 1990s, a second wave of articles appeared during recent years.

Whether epidemiological studies do or do not demonstrate an increase in autoimmune diseases is, however, less relevant when one realizes that there are patients who present with complaints that might be attributed to silicone breast implants and that explantation of the implants is an important treatment strategy to resolve these complaints.

In this review, we have therefore investigated the effectivity of explantation of the silicone breast implant in improving complaints. We found that explantation of the silicone breast implant improved silicone-related complaints in approximately 75 % of the patients (469 of 622). Autoimmune diseases improved in approximately 56 % of the patients (10 of 18); however, explantation had to be combined with immunosuppressive therapy in most of these patients.

There are several possibilities why patients improve after explantation. Two possibilities that are not mutually exclusive may clarify why explantation of breast implants may result in improvement in symptoms: (1) explantation of the silicone breast implant results in reduction in the (auto-)immune response, (2) explantation of the silicone breast implant results in reduction in nociceptive signals.

### Reduction in the immune response

In different experimental models, it has been demonstrated that silicone can induce an autoimmune or inflammatory response [38–40]. Moreover, in humans, adjacent to the periprosthetic space, chronic inflammation, characterized by proliferation of mesenchymal cells and collagen synthesis, is regularly observed. This chronic inflammation is morphologically classified as a foreign body reaction and well known as the periprosthetic capsule [41, 42]. In this periprosthetic and pericapsular space, silicone migrated from the shell of the implant can be captured by macrophages, which results in activation of these macrophages [43]. Importantly, oxygen radicals released from this activated macrophage may result in oxidation of silicone, leading to the local formation of silica [43]. In experimental studies, it is demonstrated that this results in the secretion of cytokines, promotion of fibroblast proliferation and collagen production [44]. In addition, in vivo a significant lymphoplasmatic infiltration is observed. This can lead to continuous stimulation of the immune system, leading to formation of autoantibodies and the formation of anti-silicone antibodies [5, 45–47]. It is hypothesized that the autoimmune/inflammatory process will be reduced by removing the inducing agent of this process, i.e., the silicone breast implant, [4, 10].

We observed that patients with silicone-related complaints improved after explantation, but patients who have already developed autoimmune diseases immunosuppressive drugs were additionally needed to induce remission of the disease. This implies that explantation alone is not effective for resolution of the autoimmune diseases.

An explanation for this phenomenon could be that prior to explantation silicone particles have already been migrated into the periprosthetic tissue, lymph nodes and other tissues [48, 49]. This implies that the silicone particles remain present in the body after explantation and that the autoimmune/inflammatory response continues. This could also clarify the observation that autoantibodies remain detectable after explantation.

### Reduction in nociceptive signals

Clinical findings in patients with ASIA due to SIIS resemble the clinical picture of fibromyalgia [50, 51]. Indeed, the type of complaints is more or less identical in these two diseases. It has been postulated that in fibromyalgia nociceptive signals (often psychological trauma) cause the development of symptoms via disturbed pain processing [52]. Could it be that in patients with ASIA due to SIIS the breast implant is the nociceptive stimulus causing the symptoms? Could a disturbed pain signaling pathway due to the nociceptive stimulus (silicone), in combination with extensive worrying about the safety of the breast implant, cause excessive stimulation of neurotransmitters in the central nervous system and therefore cause the systemic complaints [52]? This hypothesis may—at least partially—explain why patients experience improvement in symptoms when the silicone breast implant, and thus the nociceptive stimulus, is explanted and why improvement in the quality of life is reported. In this theory, self-evaluation after explantation/after removal of the nociceptive stimulus should improve, since there is no longer a reason for extensive worrying. More research whether the improvement after explantation is due to the removal of the nociceptive stimulus should be performed.

### Practical implications

Silicone-related complaints have been labeled differently in the past decades (human adjuvant disease, adjuvant breast disease, ASIA syndrome). Symptoms, however, did not change during the last 30 years [11]. General practitioners and other specialists should be aware that patients with silicone breast implants may present with these symptoms (Table 1). The symptoms, however, are not very specific. In making the decision that the complaints may be related to the silicone breast implants, it is therefore important to rule out other diseases. For the physician who advises the patient, it is valuable to inform the patients what the results of explantation of the breast implant might be. Importantly, explantation may results in body deformity and impaired body image, which may have a significant psychological impact [29]. Therefore, patients should also be informed what alternative reconstruction possibilities are available after explantation. Alternatives can consist of reconstruction with autologous tissue such as free flaps (LTP or DIEP flap), lipofilling (with external pre-expansion) or reconstruction with breast implants filled with saline or hydrocellulose [28, 33, 53–55]. In this review, we have found that the effect of reconstruction after explantation in patients with silicone-related complaints has received extremely little attention up until the present time. Mamma reconstruction with autologous tissue instead of implants tends to be popular in this category of patients due to a higher long-term satisfaction, higher patient’ satisfaction and a higher perception of naturalness [56]. However, in the current reviewed literature, it is not yet clear whether autologous tissue is a good alternative [33]. Reconstruction with a breast implant filled with hydrocellulose might be another alternative since exposure to silicone is diminished [15, 28]. However, the evidence for the use of hydrocellulose-filled implants as a safe alternative is at present also very limited and importantly, silicone-related complaints can also occur in patients with an implant filled with hydrocellulose or saline.

More research on which type of reconstruction could be used for patients with silicone breast implant-related complaints should be performed.

## Conclusion

The objective of this review was to investigate whether explantation of silicone breast implants in patients with silicone-related complaints is useful. We have observed that in approximately 75 % of the patients with silicone-related complaints improvement occurs. However, in patients with silicone breast implants who have developed an autoimmune disease explantation appears to be successful only when explantation is combined with immunosuppressive therapy. We postulate that both reduction in the immune response and reduction in nociceptive signals could explain why patients with silicone-related complaints experience improvement after explantation. Migration of silicone particles into the adjacent tissue could explain why explanation alone is not successful in all patients. Lastly, since very few studies dealt with the type of reconstruction for the explanted breast, we strongly suggest that more research should be done regarding this issue.
